# Successful treatment with narrowband UVB in a recalcitrant case of IgA pemphigus^☆^

**DOI:** 10.1016/j.abd.2021.09.020

**Published:** 2023-05-02

**Authors:** Lilian Lemos Costa, Roberta Buense Bedrikow, Carolina Gonçalves Contin Proença, Rute Facchini Lellis

**Affiliations:** Department of Dermatology, Santa Casa de Misericórdia de São Paulo, São Paulo, SP, Brazil

Dear Editor,

A 14-year-old female patient with a history of Down syndrome referred the appearance of lesions disseminated throughout the body that started at the age of seven. On dermatological examination, she had phototype IV skin, with erythematous plaques arranged in an annular fashion, superimposed by pustules at the periphery, located mainly on the trunk, skinfold regions, limbs and scalp, sparing the face and mucous membranes, associated with pruritus (Fig. 1). For diagnostic elucidation, a skin biopsy was performed, and histopathology showed perivascular dermatitis with subcorneal pustules. Direct immunofluorescence was positive for immunoglobulin A (IgA; Fig. 2) in an intercellular pattern and negative for immunoglobulin G (IgG). The patient was initially submitted to therapy with prednisolone at a dose of 1 mg/kg, and resolution of the condition was attained; however, after drug discontinuation, the cutaneous lesions recurred. Therapy with dapsone at a dose of 100 mg/day was then started, which she used for a period of five years, with partial control of the condition, with multiple episodes of worsening and secondary infection of the lesions.

**Fig. 1:**
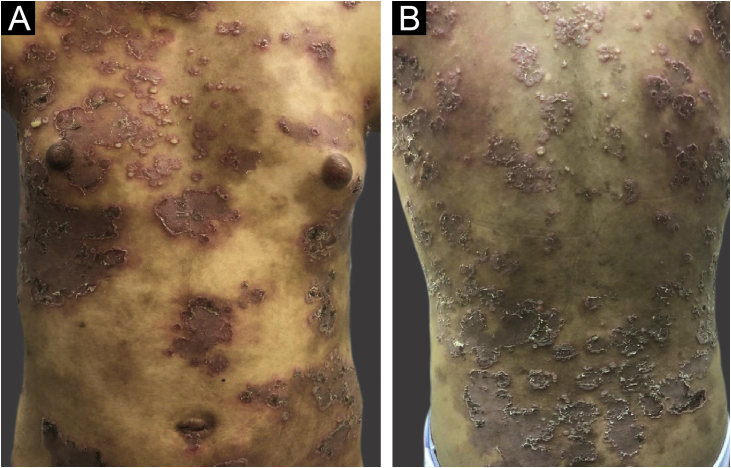
(A and B) Disseminated dermatosis characterized by circinate erythematous plaques superimposed by pustules at the periphery of the lesions

**Fig. 2:**
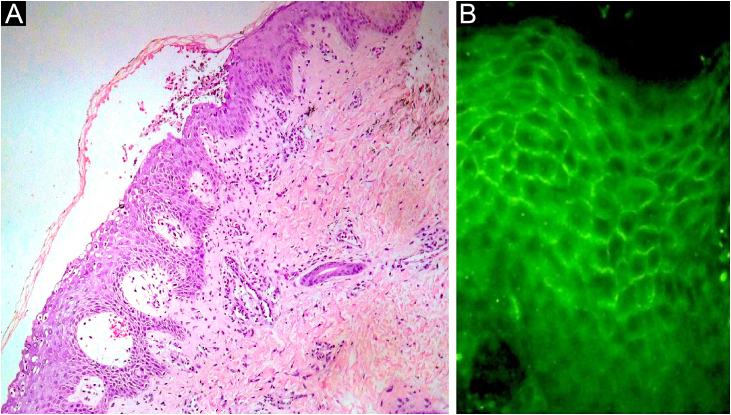
(A) Histopathology of the skin showing intraepidermal cleavage and a subcorneal pustule (Hematoxylin & eosin, ×40). (B) Positive intercellular direct immunofluorescence for IgA

She then underwent phototherapy with narrow band-UVB (NB-UVB), associated with dapsone 100 mg/day, which was already being used. After 36 sessions of NB-UVB twice a week, at a total cumulative dose of 10.7 mJ/cm^2^, the patient showed complete resolution of the skin condition, maintaining lesion remission after phototherapy discontinuation, while maintaining dapsone use. There were only residual hyperchromic macules throughout the body (Fig. 3).

**Fig. 3:**
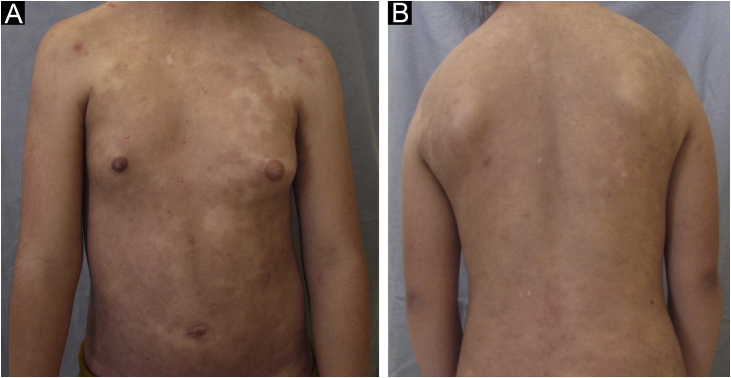
(A and B) Resolution of skin lesions after 36 sessions, 4 months of treatment, with NR-UVB phototherapy

IgA pemphigus is a rare bullous dermatosis that frequently affects adult women. This disease is uncommon in the pediatric age group.^1^, ^2^

In a descriptive study of 49 cases of IgA pemphigus, the most common forms of presentation of this disease comprised vesicles, pustules, and circinate plaques that can affect the entire body surface, but in the vast majority of cases, the presence of lesions on the trunk, extremities and intertriginous areas is frequent, as described in the present case.^2^, ^3^

Histopathologically IgA pemphigus can be divided into subcorneal pustular dermatosis and intraepidermal neutrophilic dermatosis. In a meta-analysis that assessed 136 patients diagnosed with IgA pemphigus, the subcorneal type was the most common (75.2%) and the most resistant to conventional therapies when compared to the intraepidermal neutrophilic type. In a histopathological analysis of 116 patients diagnosed with IgA pemphigus, in addition to intercellular IgA deposition, all patients had an inflammatory infiltrate in the dermis, consisting predominantly of neutrophils.^2^

The patient in the present case underwent treatment with dapsone for five years, at an optimized dose, without improvement. Therapy with NB-UVB was based on reports in the literature of its use as a therapeutic option for other chronic and immune-mediated dermatoses, being effective and safe for the treatment of children. The use of phototherapy reduces systemic therapies such as corticosteroids, and the pediatric age group presents mild, localized, and tolerable side effects.^4^

Phototherapy with NB-UVB decreases T-cell activation, the production of pro-inflammatory cytokines, and the number of antigen-presenting cells in the skin. In addition, this therapy increases the number of Treg cells that secrete suppressor cytokines, which contributes to a reduction in the inflammatory response in the dermis, a probable mechanism of action in the treatment of IgA pemphigus.^5^

There is no consensus in the literature regarding the treatment of IgA pemphigus. This study shows the use of phototherapy as a therapeutic option in the reduction of systemic therapies in extensive cutaneous dermatoses in the pediatric age group. Further studies are required to determine the mechanism of action and duration of treatment with NR-UVB to allow long-term disease control.

## Financial support

None declared.

## Authors' contributions

Lilian Lemos Costa: Approval of the final version of the manuscript; drafting and editing of the manuscript; design and planning of the study; collection, analysis and interpretation of data; critical review of the literature; critical review of the manuscript.

Roberta Buense Bedrikow: Approval of the final version of the manuscript; design and planning of the study; effective participation in research orientation; intellectual participation in the propaedeutic and/or therapeutic conduct of the studied cases; critical review of the literature; critical review of the manuscript.

Carolina Gonçalves Contin Proença: Approval of the final version of the manuscript; design and planning of the study; intellectual participation in the propaedeutic and/or therapeutic conduct of studied cases; critical review of the manuscript.

Rute Facchini Lellis: Approval of the final version of the manuscript; design and planning of the study; effective participation in research orientation; critical review of the manuscript.

## Conflicts of interest

None declared.
